# Conflicting interests, institutional fragmentation and opportunity structures: an analysis of political institutions and the health taxes regime in Pakistan

**DOI:** 10.1136/bmjgh-2023-012045

**Published:** 2023-10-16

**Authors:** Zafar Mirza, Daud Munir

**Affiliations:** 1 Professor of Health System and Population Health, School of Universal Health Coverage (Global Institute of Human Development), Shifa Tameer-e-Millat University, Islamabad, Pakistan; 2 Onto Global Ltd (A Global Development Consultancy Firm), Vancouver, British Columbia, Canada; 3 Partner, Axis Law Chambers, Islamabad, Pakistan

**Keywords:** health economics, health policy, public health, descriptive study, qualitative study

## Abstract

Pakistan is the world’s fifth most populous country, with large segments of its population at risk from non-communicable diseases caused by consumption of harmful products, including tobacco and sugar-sweetened beverages. Even though evidence exists that increased taxes on harmful products leads to consumption reductions as well as increased revenues, Pakistan’s health taxes remain low. We seek to understand the reasons for the deficient health tax regime. Much of the existing literature emphasises industry tactics, resources and motivations. We take a different approach and instead focus on political institutions in Pakistan which could help explain deficiencies in the health taxes regime. We employed a mixed method design. We conducted: (1) a detailed analysis of media content, (2) semistructured interviews with key stakeholders (and attended relevant meetings) and (3) an analysis of primary and secondary literature, including legal and policy documents. We identify two key aspects of Pakistan’s political institutions which may help explain deficiencies in health taxes. First, we identified structural issues in the design and functioning of key institutions responsible for health taxes, including with respect to federalism, intraelite conflict, interagency coordination and intra-agency fragmentation. Second, we found evidence of an entrenchment of industry interests within governmental institutions, which are characterised by weak frameworks for regulating conflicts of interest. We conclude that gaps and conflict within political institutions, owing to weak design, instability and fragmentation, create political opportunity for industry actors to influence the system to advance their interests. The findings of this research indicate towards needed interventions.

**Summary Box**

- Researchers and policy analysts have previously highlighted deficiencies in the health taxes regime in Pakistan, particularly in relation to tobacco. Previous studies have largely focused on industry tactics to explain such deficiencies.
- What this study addsThe ability of industry actors to influence policy-making depends on the nature and strength of political institutions. This study identifies gaps and weaknesses in political institutions, which create political opportunities for industry actors to advance their interests.
- How this study might affect research, practice or policyThe findings of this study suggest that there may need to be stronger recognition of underlying weaknesses in political institutions and that actual policy change may only come about through addressing such weaknesses.

## Introduction

With a population of about 230 million, Pakistan is the world’s fifth most populous country. With respect to non-communicable diseases (NCDs) linked to consumption of certain harmful products, Pakistan is among the countries with the largest numbers of people at risk.^1^

Tobacco use in Pakistan is quite high, with a variety of products consumed, including manufactured cigarettes and waterpipe (shisha), as well as chewing of *paan*, *gutka* and *naswar* (orally used snuff).^2^ According to recent estimates, 30 million adults (aged 15 and above) are currently tobacco users; this amounts to 19.1% of the population.^3^ Tobacco use is a leading cause of deaths caused by NCDs, including cardiovascular and respiratory diseases, as well as cancer.^4 5^ In the year 2017, deaths caused by tobacco were upwards of 160 000, and this number is likely to continue to grow given the number of users of tobacco also continue to increase.^6^ Research has indicated that cigarettes in Pakistan are actually becoming more affordable over time, such that per capita income required to purchase 2000 cigarettes was 4% in 2020–2021, and expected to decline to 3.2% if cigarette prices do not increase.^6^ Tobacco use places a greater burden on poor households, where spending is at the expense of basic needs.^7^

In our analysis of health taxes in Pakistan, we have focused mainly on tobacco. We note, however, that in the case of consumption of sugar, Pakistan also ranks significantly higher than comparable countries, with an estimated consumption at 25 kg per capita (compared with 19.7 kg for developing countries and 12.3 kg for least developed countries).^8 9^ Seventeen per cent of youth consume sugar-sweetened beverages daily. This has led to high rates of childhood and adolescent obesity, as well as diabetes, hypertension and heart disease in adulthood. Notably, in 2021, Pakistan ranked third in the world in terms of the number of adults (20–79 years) with diabetes, with 33 million such people with diabetes.^10^ Though we focus on tobacco taxes, we expect that the factors identified to explain low levels of taxation on tobacco will be helpful in understanding health taxes generally. Further, we have not considered alcohol because under Pakistan’s laws, subject to certain exceptions with respect to non-Muslims, there is generally a prohibition on the manufacturing, sale or possession of alcohol.

Evidence exists that health taxes can be an extremely effective mechanism to curb consumption of harmful products, including tobacco and sugar-sweetened beverages.^11–15^ However, surprisingly, taxes on harmful products such as tobacco are low in Pakistan. Currently, excise taxes on cigarettes in Pakistan are at on average approximately 45% of the retail price, significantly lower than the benchmark of 70% recommended by the WHO.^16^It has been estimated that raising excise taxes by 30% would result in both a decrease in the number of smokers (by around 200 000) as well as increasing the excise tax revenue (by around 25%) in Pakistan.^6^

One possible explanation for low taxes may be that such evidence is not known to relevant Pakistani stakeholders. This does not seem plausible since we found evidence of discussion on the matter in the public sphere, including coverage of activism and policy-based research in this regard. However, progress with respect to health taxes in general, and tobacco taxes in particular, has been very slow and the initiatives taken in this regard have normally not achieved their intended outcomes.

Previous studies attempting to understand gaps in health taxes in Pakistan have focused mainly on industry tactics.^17–23^ In other words, from this standpoint, the question related to inadequate health taxes focuses on the capabilities and tools employed by industry actors. However, this explanation possibly fails to appreciate that industry motivations, such as for the tobacco industry, are quite similar across various countries, and in most contexts, their attempt is to try and maximise profits through the use of familiar tactics. So is the case in Pakistan, where we found evidence of much of the same tactics as used in other countries^2 17^ The fact, then, that Pakistan has significantly lower health taxes on such products as tobacco, it would appear, may have something to do with national-level systems and frameworks.

Methodologically, our research takes seriously the institutional perspective within social science research.^24^ In other words, we examine the role of political institutions—or the set of structures, rules and procedures confronted by actors operating within a system—in defining and shaping the behaviour of such actors within the system. Therefore, in this article, we have analysed key institutional features within the political landscape which may help explain deficiencies in the health tax regime in Pakistan.

Our research demonstrates that there are certain features of political institutions that may be responsible for the relative efficacy of industry actors to influence the health taxes regime in the country. Through an analysis of primary and secondary data as well as semistructured interviews with key stakeholders, we found that weaknesses and gaps within political institutions may have created political opportunity structures that industry actors have been able to exploit to prevent the design and implementation of an effective health tax regime in the country.

We have used a mixed-method design in order to collect requisite information and data from a number of sources. First, we conducted a media analysis, focusing primarily on newspaper articles and reports covering health taxes and industry tactics in connection with the tobacco industry. We included in our analysis six major English language daily newspapers with a sizeable readership in Pakistan (Dawn, The News, Express Tribune, Pakistan Today, Daily Times and the Business Recorder), the articles of which are searchable using keywords (through their respective websites or through Google News). We identified and analysed 133 articles and reports from the abovementioned newspapers published in the last 10 years with the first article dated 27 March 2013. We made use of relevant key words to search for such articles, including the terms “tobacco tax”, “health tax”, “excise tax on cigarettes”, “tobacco industry”, and “conflict of interest law”. Then, we grouped the articles according to the thematic topics for analysis. Second, we conducted fourteen semistructured interviews with key stakeholders. For this purpose, our approach was to try and interview stakeholders representing various institutional perspectives, including both representatives from the senior-level government policy-makers and mid-level government officials as well as policy analysts and activists. We participated in a stakeholder forum on the issue of health taxes, from which we identified a number of the interviewees with whom we conducted the semistructured interviews. Policy analysts and civil society advocates were identified based on their published work. Before starting each of the semistructured interviews, we explained the purpose and nature of the interview and obtained the interviewee’s consent. Finally, we analysed key primary and secondary information, including legislation, regulatory frameworks and procedures, court decisions, as well as research and policy reports and articles previously published on this topic.

In terms of our methodological approach, we employ grounded theory with the purpose of generating theory based on our analysis of data collected through the above-mentioned sources.^25^ The research informing this article is based on a case study of Pakistan. We note that while case studies may not be ideal in terms of testing theories, deep analysis of a case can be of immense value in terms of generating new theory to explain key social phenomenon. With our goal being restricted to generating critical insights for purposes of attempting to theorise on this important topic, we note that a limitation of our research is that the theory generated may need to be further analysed in other contexts to test the causal impact of governance and institutional frameworks on health taxes.

Based on our research, we identified two key aspects of political institutions in Pakistan which have a nexus with the efficacy of design and implementation of health taxes in the country. First, we identified certain structural problems in the design and functioning of key institutions which seem to have prevented the implementation of an effective health tax regime. Second, we found that weak frameworks governing conflicts of interest create political opportunity structures for industry actors to influence state institutions. Results for each of these are presented below.

## Conflict and opportunity within fragmented political institutions

Our research revealed the following four issues related to political conflict and fragmentation within political institutions, which likely created political opportunities for industry actors to exert significant control over such resultingly weak institutions.

### Issues related to conflict arising from constitutional design of the federation

We found that political fragmentation arising from the design of federal institutions has had an impact on the effectiveness of policy-making in the health sector. The 2010 18th Amendment to the Constitution devolved the subject of health to the provinces. While the intent may have been improvement in the healthcare delivery system, more than a decade later, there has not been any significant improvement in health outcomes. In fact, we found that certain systemic issues have been introduced as a result of this constitutional change which remain unresolved. Most importantly, the degree of devolution in the domain of health meant that there are limited institutional mechanisms left to create a uniform national policy or regulatory framework. This became apparent especially during the COVID-19 pandemic, such that the fragmented institutional framework lacked the capacity to deal with such a health emergency, owing to the near-complete devolution of the health sector.^26^ In order to effectively handle the pandemic at the national-level, special measures had to be taken to sidestep the coordination problems inherent in the current federal design (an ad-hoc national civil-military platform, National Coordination and Operation Center, had to be established in order to effectively handle the pandemic at the national level). Policy-making in the health sector, which had already been lagging in comparison to the health crises in the country, has become increasingly more fragmented and dysfunctional. With respect to health taxes, we found that two key dimensions of the conflict arising with the federal structure are of importance.

First, the problem of political fragmentation is compounded because whereas health has now become a provincial subject, the relevant power to tax rests at the federal level. This issue surfaced in 2020 after a decision was taken by the government to impose a health tax on every pack of cigarettes.^27^Subsequently, media reports noted that, according to legal experts, such a health tax would be unconstitutional, since health was a provincial subject.^28^ The Federal Board of Revenue (FBR) itself was uncertain about the constitutionality of such a tax, and the matter was referred to the Ministry of Law & Justice. While a favourable opinion in this regard was provided by the law ministry,^29^ a prominent anti-tobacco activist we interviewed noted: “Once any concrete action is taken to levy this health tax, industry actors are highly likely to challenge the matter in court on constitutional grounds; the tobacco industry often engages the most expensive and leading lawyers to challenge tax increases, and are often able to obtain stay orders.”

Second, we learnt that a key obstacle is the nexus of federalism and party politics. Parties and coalitions who succeed at making a government at the federal level generally are not able to also form governments in all or even the majority of the provinces. With high intraelite conflict and ensuing political instability discussed in the following section, national-level policy-making by political opponents ruling various governments is often frictional rather than collaborative. For example, one of the key persons who worked on the tobacco-free cities initiative narrated during an interview that after initial success in Islamabad, when the project had to be replicated in the provinces, the team working on it thought it prudent to bypass the provincial government and work directly with the district administration, owing to political friction between the central and provincial governments in question.^30^

### Macropolitical instability and intraelite conflict

We learnt that political instability has been a contributing factor to the deficient regime for health taxes in Pakistan. At the macropolitical level, Pakistan’s democratic institutions remain fragile, and chronic political instability negatively impacts governance. Considering the last two decades, in 2008, the country returned to civilian-democratic rule after a decade of military rule. However, the nature of politics has remained highly unstable, such that no Prime Minister has completed a full term in office, or indeed during the entire history of the country. A political commentator has described the impact of such macropolitical instability on policy-making and governance as follows:

Unremitting political confrontations have left the country exhausted and emaciated… Opponents are seen as enemies, not competitors. Politics is about vanquishing the enemy and eliminating them from the political scene in a terminal conflict…. There was another cost to the country. The lack of a stable and predictable environment proved a huge hurdle to solving the country’s daunting problems, which were either left to fester or met by imprudent short-term policy responses.^31^

In the 2019–2020 budget speech, a health levy was announced on tobacco products, which was later not included in the written version of the budget speech. Subsequently, however, those favouring health taxes within the government, including the State Minister of Health, continued efforts to impose the health tax, this time through a legislation which would also ensure earmarking of the revenue for health purposes. Several tangible steps were taken in this direction, including the drafting of a ‘Health Contribution Bill’ as well as obtaining an approval from the Ministry of Law & Justice with respect to the legality of the imposition of such a tax by the Federal Government. Through interviews, we learnt that while there had been considerable progress made on this initiative, the abrupt change in government in March 2022 through a vote of no confidence by the opposition brought a complete halt to the progress.

In a context of heightened intraelite political conflict, even during periods of civilian democratic rule, the terms of debate have not been focused on critical areas of policy-making such as health, education and poverty eradication. Instead, with the government’s effort to retain power and the opposition’s efforts to prematurely end the ongoing term, politics has remained focused on denigrating opponents often in connection with political scandals and corrupt practices, rather than critically analysing and opposing policy-making agendas. Further, when governments or leaders are not able to complete their terms, successors often do not have the preparation or enough time remaining to deliver on demanding and time-consuming policy-making agendas, such as bringing new legislation. As the example above demonstrates, this overall weakness in political institutions and governance caused by political instability opens up a political opportunity structure for highly cohesive industry actors to influence the system.

### Lack of interagency coordination and conflicting interests

We also found evidence of friction and lack of coordination among federal agencies responsible for health policy and taxes. A large number of ministries and agencies are involved in the decision-making process, including the ministries of finance, health, commerce, as well as specialised bodies such as the FBR and the Pakistan Tobacco Board. Each of these agencies has their own mandate and focus, which are in some cases diametrically opposed. In particular, it is noteworthy that the Federal Government has set up the Pakistan Tobacco Board, a specialised agency whose mandate is to promote the tobacco industry. Hence, we saw evidence of an inherent conflict and fragmentation in the institutional design with respect to this issue, such that proposals from the Ministry of Health have a direct conflict with the mandate of the Pakistan Tobacco Board. We learnt that with respect to tobacco taxes, though health ministry often generates proposals, it does not seem to have the requisite authority to ultimately push such proposals towards implementation. As a policy matter, the ultimate decision is made by the government through the federal cabinet and/or the parliament and it is implemented through the FBR which is under the ministry of finance. However, as is the case with certain other areas of public policy, there is no overarching authority or committee empowered to make decisions in this regard. In this context, a former health minister commenting on a stalled attempt at greater regulation of the tobacco industry stated that since the industry is one of the biggest taxpayers, the finance ministry and FBR have ‘some soft corners for them’.^32^As a result of the overall fragmentation, industry actors have a political opportunity structure with openings available at various places within the system for exerting influence.

### Issues related to intra-agency fragmentation

Other than macrolevel political instability discussed above, we also found evidence of microlevel instability at the institutional level, which seems to have negative impact with respect to the ability of key institutions to deliver. One of the most important structural aspects we found was with respect to effectiveness of leadership. We found frequent changes in the heads of key institutions, which likely had strong negative impacts on effectiveness and continuity in policy-making and implementation. For example, in the last 5 years, Pakistan has had five different health ministers. Again, in the last 5 years, there have been 10 different chairpersons of the FBR. Further, federal secretaries, who act as the administrative heads of government ministries, are changed frequently, often serving in a single ministry for less than 6 months. This trend (of ad-hocism) shows that, without security of tenure, taking tough policy decisions against entrenched interests may be especially difficult, since the likely consequence may be removal from office or transfer to another position. These findings are consistent with research in other countries which indicate a negative impact of short tenures on healthcare systems.^33^

Further, amid political uncertainty and instability, there are issues related to continuity of programmes and initiatives. We found, for example, that in April 2021 the government abruptly decided to disband the Tobacco Control Cell that had been set up within the federal health ministry in 2007 with a mandate to control tobacco use in Pakistan.^34^ This was possible because the cell was setup in an ad-hoc manner within the ministry, rather than having been established on more solid legal foundations. Through interviews and media reports, we learnt that tobacco industry had not been comfortable with such special unit existing within the ministry, and had exerted its influence to abolish the cell.

## Entrenched interests and frameworks governing conflicts of interest

One of our most important findings was the entrenchment of industry interests, directly and indirectly, within governmental institutions. For example, as also mentioned below, we learnt through numerous media reports as well interviews with key informants that the owner of a local tobacco company, who is also a member of the Senate of Pakistan, has served in consequential roles related to the tobacco industry. It is noteworthy that the said senator’s connection with the tobacco industry is not an unknown fact. It was reported in July 2020 that special cigarette packets with the brand name ‘Senate House’ were distributed free by the aforementioned senator to other members of the Senate.^35^ It is noteworthy that the cigarette packs had Senate House written on them, and also included a ‘Government of Pakistan’ emblem on the pack. While analysts concluded that the senator’s action in distributing this new brand of cigarettes violated a number of laws, including related to mandatory health warnings, distribution of free samples and wrongful appropriation of official emblems; such a serious violation did not have any meaningful consequences for the legislator and others responsible for the violation.

Tracing the impact of conflicts of interest is often difficult, as such conflict is often known only to the conflicted party and operates through subtle mechanisms.^36^ However, analysis of certain more visible cases may make apparent the nature and extent of such influence. One such example is of the appointment of above-mentioned senator as member of FBR’s Policy Board, the policy-making body which plays a highly consequential role with respect to the determination of tobacco taxes.^37^ Further, in another instance of direct conflict of interest, the same senator was also made a member of a special committee of the Senate of Pakistan on ‘Causes of Decline in Tax Collection in Tobacco Sector’.^38^

The entrenchment of industry actors in governmental institutions spans across political parties as well as various branches and agencies. For example, in July 2022, a parliamentarian wrote a letter to the Cabinet Division alleging that a sitting minister had been openly lobbying on behalf of the tobacco industry, despite her husband being an employee of one large national tobacco company.^39^ The parliamentarian alleged that the minister attended meetings where such matters were taken up, and where she would otherwise not be expected to be present. In terms of entrenchment in key institutions within the executive, during interviews key informants noted that relatives of senior FBR officials have been known to be employed by tobacco companies. In this regard, we learnt that when a probe had been conducted through a parliamentary committee in 2018, the FBR adopted dilatory tactics with respect to the submission of the required information. Same is the case with the entrenchment of sugar industry actors within various governmental institutions, including the legislature. In 2020, a specially constituted Sugar Inquiry Commission submitted its findings to the Federal Government, which revealed the powerful influence of sugar industry actors within the government.^40^

Notably, such entrenchment of industry players seems to have had a significant negative impact on the health tax regime. For example, a previous research study reported that a leading tobacco company hired the husband of a federal minister to facilitate a 30% reduction in tax rates for a period of 2 years.^17^ Further, in June 2020, an advance tax on tobacco was removed by the government after lobbying efforts including involving politicians with direct and indirect interests in tobacco farming, manufacturing and trade. Another example from 2020 is when the Federal Cabinet approved a health tax on cigarettes which was included in the budget speech, but owing to the ability of the industry to influence from within, the item was dropped from the written version of the speech later made public. However, more than such reversals, the real problem lies with the weak support for health taxes in the first place, owing to which several initiatives never see the light of day. For example, in 2018, a serious attempt had been made to reform the Green Leaf Threshing tax regime, and despite tangible steps taken in this direction, these reforms were not ultimately approved owing to the ability of tobacco manufacturers to influence the government at high levels, including from within.^17^

The above-mentioned examples need to be explored in the context of the effectiveness of the political institutions in place to manage and govern conflicts of interest. Our research revealed significant gaps in the legal framework governing conflict of interest for both legislators and public servants in Pakistan. With respect to the legislative branch, for example, the Rules of Procedure and Conduct of Business in the National Assembly, 2007, restrain a member from voting on matters in which they have a pecuniary interest. However, an explanation has been inserted in said rule which states that the ‘pecuniary interest contemplated in this sub-rule shall be direct and personal and not remote or general’.^41^As noted above, such rules have not prevented involvement of industry players even when they were direct beneficiaries (as owners or directors of companies), but as drafted, it appears that the rule is not even intended to cover instances in which family members or other relations would stand to benefit. An anti-tobacco advocate we interviewed noted: “The businessmen get themselves elected to support their businesses, and once in the assemblies, try to get themselves nominated to relevant committees by claiming subject-matter expertise; once on key positions however, they behave less like parliamentarians and more like lobbyists.”

With respect to the executive, we note there is currently no legal framework in place that specifically deals with regulation of conflicts of interest for public officials. We found that there have been a few unsuccessful attempts to pass such a law. In 2016, the National Conflict of Interest Bill was tabled before the National Assembly, but the bill was rejected by the concerned Standing Committee.^42^ In 2020, another effort was made by the then Prime Minister and his Cabinet to prepare a law regarding conflict of interest, but this effort also did not succeed.^43^ While there are some other laws which may have some relevance to management of conflict of interest, overall the regime remains deficient. Further, our research did not reveal any robust conflict of interest policies in place at key agencies dealing with health taxes. For example, analysis of publicly available policies of the FBR showed that the conflict of interest provisions in such policies focused exclusively on preventing FBR officials from preparation of taxes for other individuals, and lacked provisions focused on management of conflicts of interest.

## Conclusion

We depart from previous studies on health taxes in Pakistan which place primary emphasis on industry tactics in explaining the relative efficacy of health taxes. We find that industry actors employ similar tactics, are often resourceful and collaborative, and are motivated by the same logic across countries.^44^ This is particularly the case with the tobacco industry, which is dominated globally by certain multinational corporations. To understand the differential success of industry actors, it appears, we must look at the governance interface. Political institutions, including structures, rules and procedures operate much like an immune system in terms of protecting the public from the agendas of cohesive actors who may attempt to influence policy-making in directions not favourable for the public.

With respect to the above approach, one may ask whether institutions actually matter, and whether they have any autonomous role to play in the regulation of political behaviour. While there is credible evidence in this regard in political science literature,^45 46^ with respect to the present case, we note that reform of political institutions has previously led to actual change in this domain. For example, the tobacco-free cities initiative, as well as the banning of *shisha* bars in Pakistan, have been relative success stories, whereby changes in legal regimes translated into actual change in conduct.^47^ What may differentiate these examples from the issue of health taxes is that in the case of smoking in public places (including in connection with outlawing of *shisha* bars) the main affected parties were restaurants and cafes, which face the collective action problem and are not as resourceful and cohesive as the tobacco and sugar-sweetened beverage industries. However, the core point still remains: transformation of structures and rules led to successes in compliance and change in conduct.

A focus on political institutions is of much importance, from a policy perspective. With respect to public health research and advocacy focused on harmful products such as tobacco and sugar-sweetened beverages, such a focus may lead to a realignment in the approach towards affecting policy change. We found, for example, that policy analysts and activists had taken a number of actions in the public sphere to try and highlight the importance of health taxes, as well as the industry tactics to thwart their imposition and implementation. However, if indeed the problem lies with the weakness in political institutions, then policy analysis and activism would need to be realigned accordingly.

We find three such areas of focus. First, given the large political opportunity structure available to the industry because of a virtually non-existent regime for regulating conflicts of interest, policy analysts and activists may need to make the strengthening of the institutional framework for governing conflicts of interest a central goal.^36^ While this would be a cross-cutting measure involving a large number of sectors, the importance of effective frameworks for managing conflicts of interest and corruption in health systems is of fundamental importance in terms of achieving healthcare reforms and eventually outcomes.^48 49^ Second, given endemic instability within political institutions, reform measures will have to be designed keeping in view an environment of uncertainty. Finally, rather than a direct focus on industry tactics only, a more central focus will need to be brought to the design and functioning of key institutions responsible for health taxes. For example, research has shown that at least some degree of policy-making or regulatory capacity at the central level may be important for good governance with respect to health and other social subjects.^50 51^ Therefore, the precise allocation of the various aspects of the subject of health at the national and subnational levels would need further analysis. Further, with respect to lack of interagency coordination, it may be helpful to consider solutions, such as the proposal to set up a new authority focused on tobacco control, which would have strong powers under legislation for taking effective measures including through implementing health taxes.^52^ Thinking of the various microlevel issues highlighted above, which need to be resolved for a number of the institutions, the proposed new agency could have a robust conflict of interest framework, as well as security of tenure for its leadership and employees to offer protection against industry influence.

The design and functioning of political institutions, including the ability of government to deliver, are vast areas of research. Our contribution in this article was to focus specifically on the issue of health taxes, in order to analyse the political institutions and features thereof which may be most relevant in terms of governance failures leading to ineffective tax regimes. We conclude that gaps and conflict within political institutions, owing to weak design, instability, and fragmentation, open up political opportunity structures for cohesive industry actors to influence the system relatively more effectively than in other contexts with more robust institutions and better governance.

In examining the gaps and weaknesses of the governance framework regulating health taxes in Pakistan, we do not overlook incremental progress and certain substantial initiatives that have been taken from time to time. One recent such example is the track and trace system being gradually implemented by the FBR to combat counterfeiting and to prevent evasion of federal excise duty.^53^ Further, notable actions have been taken by civil society advocates as well as by champions within the government to try and increase taxes on harmful products (or, as in the case of *shisha* cited above, to impose a complete ban on such activity in public places). From our institutional analysis, we note, however, that there are certain structural factors that are likely to interfere with the effectiveness of public policy initiatives such as imposition of adequate health taxes. Therefore, alongside analysis and advocacy with respect to harmful products and taxing of such products, a focus on certain deeper institutional factors will be critical in terms of encouraging a more effective implementation of policy tools such as health taxes.

## Data Availability

Data are available upon reasonable request.
